# Unraveling the Mystery Surrounding Post-Acute Sequelae of COVID-19

**DOI:** 10.3389/fimmu.2021.686029

**Published:** 2021-06-30

**Authors:** Rakhee K. Ramakrishnan, Tarek Kashour, Qutayba Hamid, Rabih Halwani, Imad M. Tleyjeh

**Affiliations:** ^1^ College of Medicine, University of Sharjah, Sharjah, United Arab Emirates; ^2^ Sharjah Institute for Medical Research, University of Sharjah, Sharjah, United Arab Emirates; ^3^ Department of Cardiac Sciences, King Fahad Cardiac Center, King Saud University Medical City, King Saud University, Riyadh, Saudi Arabia; ^4^ Meakins-Christie Laboratories, Research Institute of the McGill University Healthy Center, McGill University, Montreal, QC, Canada; ^5^ Prince Abdullah Ben Khaled Celiac Disease Chair, Department of Pediatrics, Faculty of Medicine, King Saud University, Riyadh, Saudi Arabia; ^6^ Infectious Diseases Section, Department of Medical Specialties, King Fahad Medical City, Riyadh, Saudi Arabia; ^7^ College of Medicine, Alfaisal University, Riyadh, Saudi Arabia; ^8^ Division of Infectious Diseases, Mayo Clinic College of Medicine and Science, Rochester, MN, United States; ^9^ Division of Epidemiology, Mayo Clinic College of Medicine and Science, Rochester, MN, United States

**Keywords:** long COVID-19, chronic COVID-19, long-haulers, SARS-CoV-2, clinical manifestations, immunopathology, post-acute COVID-19 syndrome, post-acute sequelae of COVID-19

## Abstract

More than one year since its emergence, corona virus disease 2019 (COVID-19) is still looming large with a paucity of treatment options. To add to this burden, a sizeable subset of patients who have recovered from acute COVID-19 infection have reported lingering symptoms, leading to significant disability and impairment of their daily life activities. These patients are considered to suffer from what has been termed as “chronic” or “long” COVID-19 or a form of post-acute sequelae of COVID-19, and patients experiencing this syndrome have been termed COVID-19 long-haulers. Despite recovery from infection, the persistence of atypical chronic symptoms, including extreme fatigue, shortness of breath, joint pains, brain fogs, anxiety and depression, that could last for months implies an underlying disease pathology that persist beyond the acute presentation of the disease. As opposed to the direct effects of the virus itself, the immune response to severe acute respiratory syndrome coronavirus 2 (SARS-CoV-2) is believed to be largely responsible for the appearance of these lasting symptoms, possibly through facilitating an ongoing inflammatory process. In this review, we hypothesize potential immunological mechanisms underlying these persistent and prolonged effects, and describe the multi-organ long-term manifestations of COVID-19.

## Introduction

With the corona virus disease 2019 (COVID-19) still looming large, it is becoming increasingly evident that the severe acute respiratory syndrome coronavirus 2 (SARS-CoV-2) is accountable for previously unexpected long-term health consequences. Unlike originally thought, the novel SARS-CoV-2 virus affects not just the lungs but multiple organ systems, including heart, brain, liver, kidneys and gastrointestinal tract. While the majority of patients are either asymptomatic or present with mild disease that recovers within a couple of weeks, some patients with severe disease require hospitalization and intensive care with associated mortality. At the same time, clinicians are increasingly seeing cases of those who exhibit chronic, diverse and ongoing disease symptoms even after their initial recovery from viral infection.

Critical illnesses are known to be causally associated with longer-term outcomes. Likewise, chronic cases of COVID-19, especially those with lingering disease collectively termed as “long-haulers”, are re-defining the whole COVID-19 pandemic. A wide array of names is used to refer to this subset of COVID-19 survivors, including long COVID, chronic COVID, post-acute COVID syndrome, post-acute COVID-19, long-term effects of COVID, long-haul COVID, late sequelae, as well as the research terminology post-acute sequalae of COVID-19 or SARS-COV-2 infection (1).

**Figure 1:**
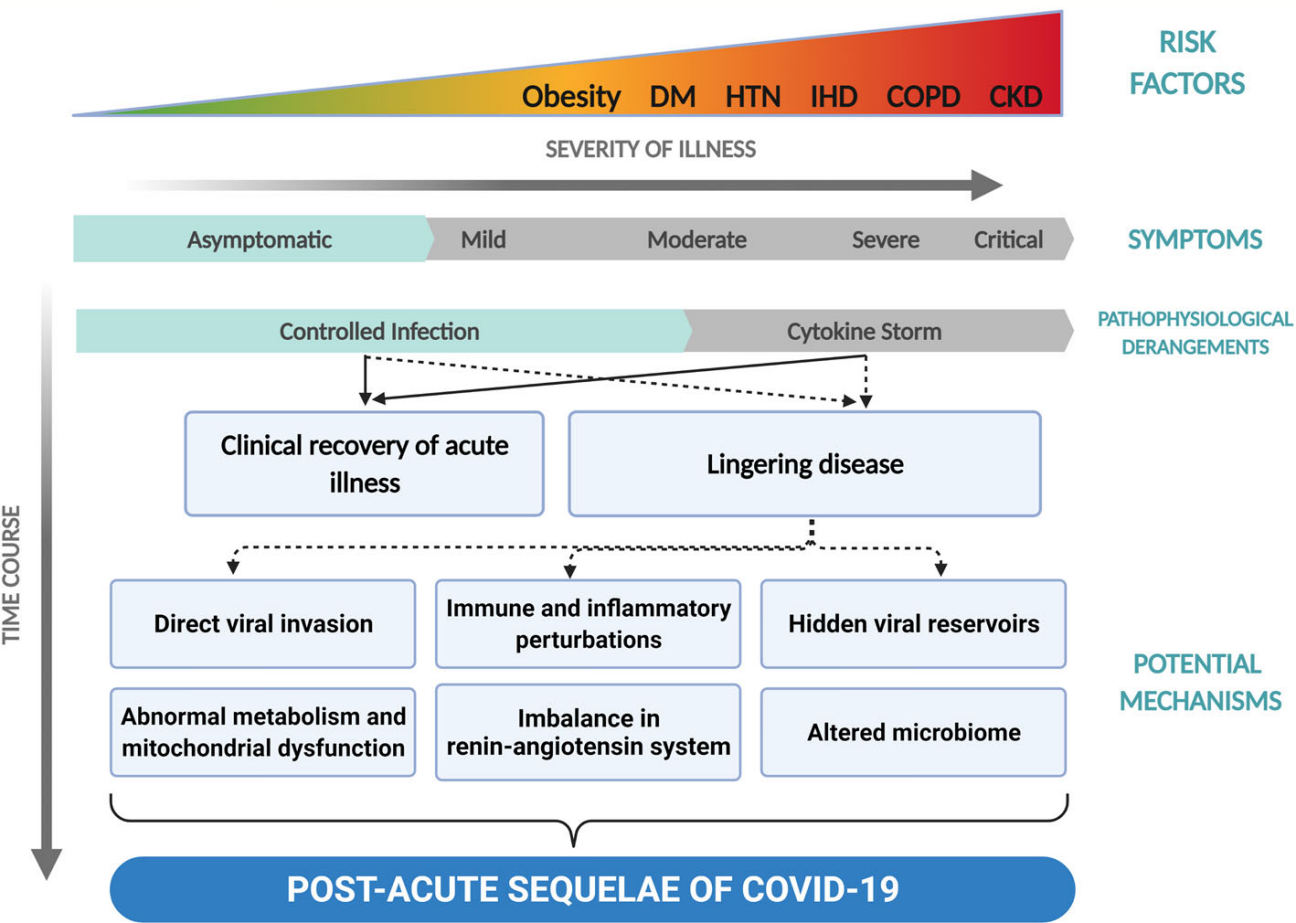
Development of post-acute sequelae of COVID-19 (PASC). This figure illustrates the development of PASC with time across the spectrum of severities presented by COVID-19. DM, diabetes mellitus; HTN, hypertension; IHD, ischemic heart disease; COPD, chronic obstructive pulmonary disease; CKD, chronic kidney disease.

A sizeable subset of COVID-19 patients continues to experience chronic persistent symptoms even after recovery from acute infection, such as extreme fatigue, shortness of breath, joint pains, brain fogs and mood swings (2–4). These symptoms persist beyond the acute presentation of the disease and appear to be independent of disease severity (**Figure 1**). These long-haulers present with a disease portrait distinct from the typical acute COVID-19 disease. They broadly represent patients who have failed to return to their baseline health post-acute COVID-19 infection.

The chronic symptoms in these patients are suggestive of ongoing pathophysiological processes post-COVID-19 infection. Besides the virus itself, the immune response to SARS-CoV-2 could be held responsible for the appearance of these lasting symptoms, possibly through triggering an ongoing inflammatory process (**Figure 1**). The diverse symptomatic manifestations of post-acute COVID-19, the potential underlying immunopathological mechanisms and evidence of multi-organ clinical sequelae of COVID-19 will be discussed in this review.

## Definition of Post-Acute COVID-19

With increasing scientific and clinical evidence on the long-term outcomes of COVID-19, the definition of post-acute COVID-19 has evolved along the way. Although there is no consensus on the definition yet, a group of investigators have defined it as the persistence of symptoms extending beyond 12 weeks from initial symptom onset (5). In the absence of standardized guidelines and recommendations on post-acute COVID-19, the National Institute for Health and Care Excellence (NICE) in collaboration with the Scottish Intercollegiate Guidelines Network (SIGN) and the Royal College of General Practitioners (RCGP) developed a rapid guideline on long COVID-19 aimed at identifying, assessing and managing the long-term effects of COVID-19 (6, 7). This serves as a living guideline which will be updated regularly as new evidence emerges. As per this guidance, the varying courses of COVID-19 infection were assigned the following clinical definitions:

“Acute COVID-19: signs and symptoms of COVID-19 for up to 4 weeks; Ongoing symptomatic COVID-19: signs and symptoms of COVID-19 from 4 to 12 weeks; Post-COVID-19 syndrome: signs and symptoms that develop during or after an infection consistent with COVID-19, continue for more than 12 weeks and are not explained by an alternative diagnosis” (6).

For the purpose of this review, the terminology post-acute sequalae of COVID-19 (PASC) will be used henceforth.

## Prevalence and Incidence of Post-Acute Sequelae of COVID-19

Multiple clinical studies have identified the prevalence of lingering damage in COVID-19 survivors post hospitalization across the United States (US) (8, 9), Asia (10) and Europe (3, 11–13). Laboratory-confirmed COVID-19 patients were found to experience worsening respiratory status, physical and mental health more than one month after hospital discharge compared to their pre-COVID state (14). Symptoms have been reported to persist for 2 months after onset and post-discharge from COVID-19 hospitalization (3, 15). In an observational cohort study from the US, ongoing morbidity was reported in 32.6% of 488 COVID-19 patients at 60 days after discharge (8). At 6 months post-acute SARS-CoV-2 infection, more than 70% of a large Chinese cohort experienced at least one symptom (10). Furthermore, in an Italian study, only 12.6% of the 143 patient cohort discharged from COVID-19 hospitalization were reported to be completely free from symptoms after a mean of 60 days after onset of symptoms (3). In addition to the critically ill and hospitalized COVID-19 patients, outpatients with mild COVID-19 were observed to experience a delayed return to their usual baseline health (16). This suggests a prolonged illness even among young adults with mild or asymptomatic disease. Further, the emergence of Multisystem Inflammatory Syndrome in Children (MIS-C) reinforces the presence of post-acute manifestations of COVID-19 across all age groups (17). The rapid increase in the number of reports on PASC is indeed alarming with these severe and long-term after-effects of SARS-CoV-2 infection extending the stakes of the pandemic beyond what we once thought.

## Manifestation of Symptoms in Post-Acute Sequelae of COVID-19

Patients with post-acute sequalae of COVID-19 (PASC) develop significant limitations in activities of daily living (ADLs) such as walking, bathing, or dressing with multi-factorial causes of this functional decline (18). This physical weakness can be attributed to myopathy, neuropathy, cardio-respiratory impairments, cognitive impairment, or a combination of these conditions. COVID-19 long-haulers thus, show multiple diverse symptoms affecting different parts of the body, such as nausea, extreme fatigue, dyspnea or shortness of breath, cough, chest pain, brain fog, short-term memory loss, palpitations, excessive bruising, joint pain, light and sound sensitivity, coagulation, neurological, gastrointestinal and gynecological problems (3, 19). These lingering chronic symptoms reflect damage across multiple organs. A recent study that investigated the long-term health consequences of COVID-19 in patients discharged from hospital found fatigue or muscle weakness in 63%, sleep difficulties in 26%, and anxiety or depression in 23% of the cohort (10). In this study, the patients with severities during their hospital stay demonstrated more severe impaired pulmonary diffusion capacities and abnormal chest imaging manifestations. Additionally, dermatological manifestations in PASC included papulosquamous eruptions, in particular pernio- or chilblain-like lesions (20). Neurological manifestations, such as impaired consciousness, acute cerebrovascular diseases and skeletal muscle injury, were demonstrated in COVID-19 patients with more severe infection (21). In addition to posing as risk factors for the disease itself, factors such as age, frailty and presence of comorbidities, they also modulate the long-term impact of the disease.

Interestingly, similar observations of long-term sequelae has been reported following the SARS-CoV-1 outbreak of 2003 (22), and Middle East Respiratory Syndrome (MERS) outbreak of 2012 as well (23). The survivors from the previous two CoV epidemics were found to experience persistent shortness of breath, fatigue, mental health problems and reduced quality of life that constituted a kind of post-acute viral syndrome (23). A one-year follow-up study of SARS survivors reported a significant reduction in mental health (24). Another 15-year follow-up study showed partially reversible femoral head necrosis, pulmonary interstitial damage and associated functional decline that remained stable after an initial improvement in SARS patients (25). In addition, chronic widespread musculoskeletal pain, persistent fatigue, weakness, depression and disordered sleep characterized chronic post-SARS syndrome (26). These characteristic symptoms overlapped with those observed in chronic fatigue syndrome and fibromyalgia syndrome (26). Similarly, MERS survivors reported reduced overall quality of life and quality of physical health at approximately 14 months of follow-up (27). As seen with SARS survivors, high levels of chronic fatigue symptoms and psychiatric disorders, including post-traumatic stress disorder (PTSD), anxiety, and depression, were noted at one-year follow-up of MERS patients (28). Intriguingly, similar findings were also observed among non-CoV-related ARDS survivors (29) and in those who required critical care (30). These post-acute variants of COVID-19, SARS and MERS thus show similarities to other illnesses, such as chronic fatigue syndrome (myalgic encephalomyelitis), sepsis and dysautonomia.

Collectively, dyspnea, fatigue, sleep disorders and psychological issues, including anxiety, depression, PTSD and concentration problems, constituted the most commonly reported persistent symptoms across majority of the COVID-19 study participants at follow-up. These clinical manifestations of PASC could be a result of viral invasion directly into the tissues possibly facilitated by its receptor angiotensin-converting enzyme 2 (ACE2) expression, immune system dysregulation, hyperinflammation and cytokine storm syndrome, immune-mediated multi-system damage, maladaptation of ACE2-related pathways, endothelial damage and microvascular injury, hypercoagulable states associated with COVID-19, critical care-associated sequelae or a combination of these (31, 32). Since dysregulation of immune response is central to the etiopathogenesis of the acute phase of COVID-19, we hypothesize possible immunopathological mechanisms that may contribute to the pathophysiology of post-acute sequelae of COVID-19.

## Potential Immunopathological Mechanisms Underlying Post-Acute Sequelae of COVID-19

**Figure 2:**
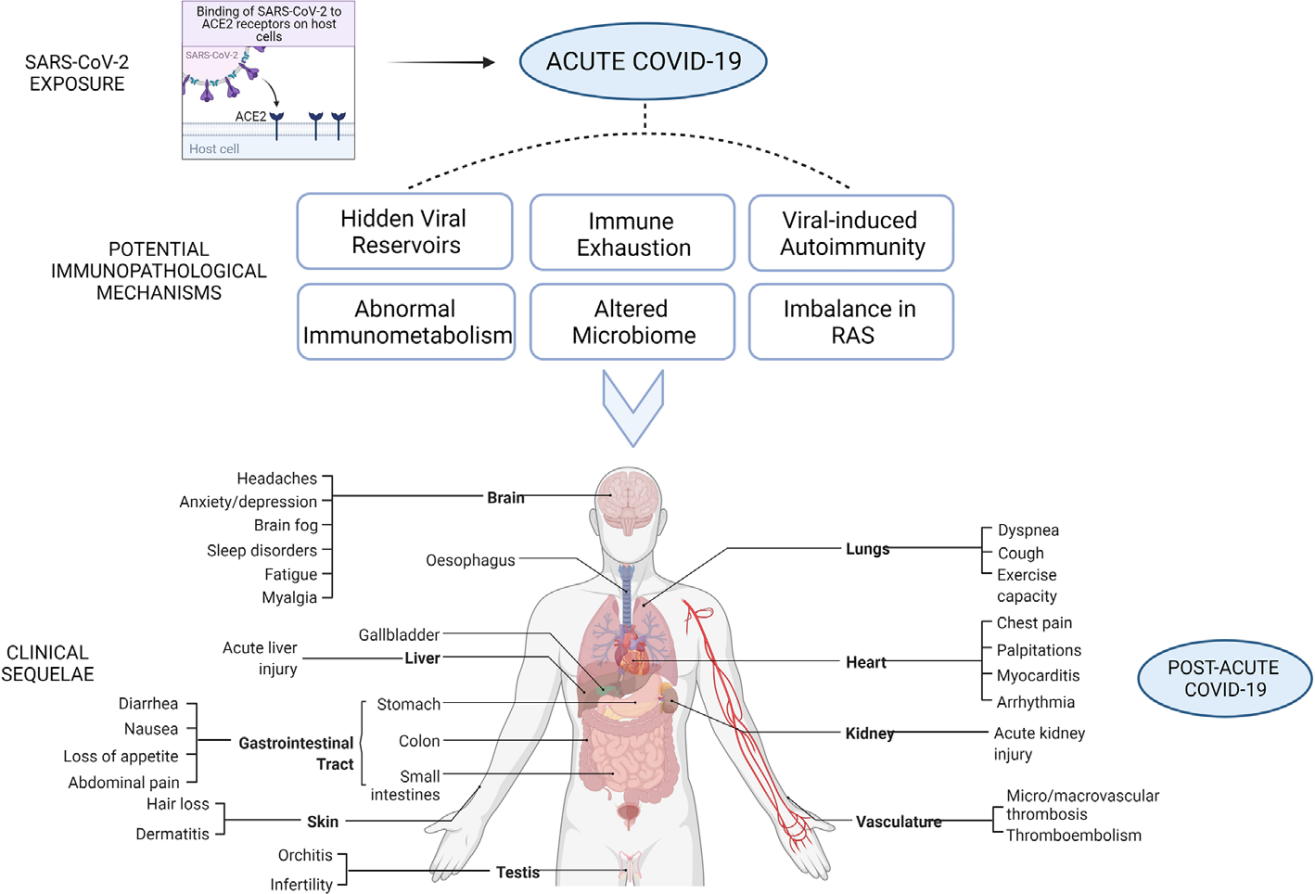
Potential immunopathological mechanims underlying multi-organ sequelae of post-acute sequelae of COVID-19 (PASC).

Aberrant cellular or humoral immune responses are believed to be the primary culprit responsible for the lasting symptoms associated with PASC. Herein, we summarize several hypotheses backed by literature to explain the long-term outcomes of COVID-19 infection (**Figure 2**): a) COVID-19 survivors with persistent symptoms may harbor the virus in several potential tissue reservoirs across the body, which may not be identified by nasopharyngeal swabs, b) delayed viral clearance due to immune exhaustion resulting in chronic inflammation and impaired tissue repair, c) cross reactivity of SARS-CoV-2-specific antibodies with host proteins resulting in autoimmunity, d) mitochondrial dysfunction and impaired immunometabolism, e) alterations in microbiome, and f) imbalance in renin-angiotensin system leading to the long-term health consequences of COVID-19.

### Hidden Viral Reservoirs & Non-Infectious Viral Fragments

The lingering disease symptoms post viral infection may relate to the significant molecular and cellular damage caused by the virus. This widespread damage in turn can be due to the wide distribution pattern of ACE2 (33), the cellular entry receptor for SARS-CoV-2 (34), and to the indirect effects of the inflammatory mediators triggered by the virus. One study provided evidence for the persistence of residual virus in the lungs, suggesting the likelihood of SARS-CoV-2 virus to cause lasting lung damage (35). Although SARS-CoV-2 preferentially infects the respiratory tract (36), the broad organotropism of this virus is fast emerging. For instance, one autopsy study of 22 COVID-19 patients detected viral presence in multiple organs, including the heart, brain, liver, kidneys and blood, in addition to the respiratory system (33). In another study of 14 asymptomatic patients that underwent small intestinal biopsies 4 months after COVID-19 onset, persistent SARS-CoV-2 was detected in half of them (37). As such, several studies have shown the presence of gastrointestinal reservoirs of infectivity post viral infection (37–39). Human body fluids, including bronchoalveolar lavage, sputum, saliva, blood, urine, feces, tears, and semen, from organs expressing the ACE2 receptor may also harbor the virus (40, 41). This multiorgan tropism is a potential explanation for the lingering long-term symptoms of COVID-19, where the virus or viral fragments could hide in reservoirs beyond the respiratory tract and hence go undetected in a nasal swab test. Another evidence in support of the reservoir theory is the observation of long-term SARS-CoV-2 viral shedding among COVID-19 patients, which extended over 3 months in some patients (42, 43). This viral persistence could then trigger chronic inflammation which may underlie the chronic COVID-19 symptoms. A similar pattern has been observed in chronic viral arthritis caused by the chikungunya virus (44), where viral RNA was found to persist in myofibers, dermal and muscle fibroblasts resulting in chronic musculoskeletal pain months to years post the initial acute infection (45). Additionally, there also exists the possibility that these reservoirs could be established in immune-privileged sites that are less accessible to the immune system such as the eye, testes, placenta, brain and central nervous system, resulting in festering infection (46). Several SARS-CoV-2 proteins have high homology with similar proteins of SARS-CoV-1 virus (47), which have been shown to play a role in evasion of the immune system (48).The viral organ reservoirs could thus go unnoticed possibly leading to the establishment of a chronic disease state.

A recent study also hinted at an unexpected hiding spot for fragments of SARS-CoV-2 genetic material within human chromosomes (49). Chimeric transcripts comprising of viral sequences fused to cellular sequences were detected in published data sets of SARS-CoV-2-infected cultured cells and patient-derived tissues. The possibility of SARS-CoV-2 retro-integration into human cellular genome and subsequent transcription of these integrated viral sequences was demonstrated in this study. This may explain SARS-CoV-2 PCR positivity in COVID-19 survivors in the absence of true infection.

SARS-CoV-2 re-infection in COVID-19 patients is another matter of emerging concern (50). SARS-CoV-2 being a novel coronavirus, the duration of protective immunity to re-infection is largely uncertain (51). To add to this, the genetic makeup of the re-infected viral strain was found to be divergent from the original one (52) and re-infection could be associated with more severe outcomes (53). The original virus hiding away in tissues and undergoing mutations could offer a potential explanation by leading to a viral strain with a different genetic background. This different strain could be responsible for re-infection or rather a reactivation of the pre-existing virus, as observed in the case of human immunodeficiency virus (HIV) (54, 55). The residual low level of virus may be responsible for at least some of the clinical manifestations of PASC, such as the persistent loss of smell and taste (55). Viral persistence and its associated genetic mutations are capable of provoking anti-viral “antibody waves” leading to immune exhaustion which may further explain SARS-CoV-2 re-infections.

### Chronic COVID-19 Associated Immune Exhaustion

Immune exhaustion is a phenomenon that is frequently associated with chronic viral infections. Immune exhaustion is defined as the dysfunction of antigen-specific immune cells due to prolonged antigen stimulation (56). The main features of T cell exhaustion include reduced cytokine production, lack of clonal expansion, upregulation of co-inhibitory receptors, altered metabolism, impaired proliferation and memory cell response (57, 58). High levels of IL-10 and TGF-β have been shown to enhance T cell exhaustion (59). This exhaustion of T cells is believed to contribute to SARS-CoV-2 viral persistence. Notably, a marked decrease in the absolute number and functional exhaustion of anti-viral cytotoxic lymphocytes, including cytotoxic T lymphocytes (CTLs) and natural killer (NK) cells, were reported in patients with SARS-CoV-2 infection, particularly in those with severe disease (60, 61). Furthermore, severe impairment of T cell subtypes, including naïve, effector memory, central memory, terminally differentiated and regulatory cells, which expressed markers of cellular exhaustion was noted in COVID-19 patients (62). In fact, our group has recently shown that a large number of immunoinhibitory receptors (IRs) expressed on both lymphoid and myeloid cells were upregulated during COVID-19 infection (63). The gene expression of eight lymphoid-associated IRs (BTLA, LAG3, FCGR2B, PDCD1, CEACAM1, CTLA4, CD72, and SIGLEC7) was specifically upregulated in autopsies, reflecting severe, terminal stage COVID-19 disease; and their expression correlated with viral levels in lung tissue. In addition, compared to SARS-CoV-1, influenza and respiratory syncytial virus infections, the number and intensities of the upregulated IRs were higher during SARS-CoV-2 infections. Moreover, an escalation in the expression of exhaustion modules, including PD-1, CTLA-4, TIGIT and Tim-3, was observed in both lung-resident and circulating T cells from COVID-19 patients (61, 64). Besides, an increased population of lung-infiltrating CD8+ T cells exhibiting transcriptional hallmarks of terminal T cell exhaustion, including expression of *CCL4, GZMB, MK167* and *TYMS*, was detected in severe COVID-19 patients (65). Thus, similar to other chronic viral infections, SARS-CoV-2 appears to severely impair the functional subsets of CD4+ and CD8+ T cells. Thus, immune exhaustion could facilitate SARS-CoV-2 dissemination, which could in turn have long-term consequences resulting in PASC.

At the same time, it is important to not ignore the contribution of T cells to inflammation and associated lung injury. SARS-CoV-2 may possess superantigens capable of binding MHCII and acting as potent polyclonal T cell mitogens (66). This could be a possible explanation for the toxic shock syndrome exhibited by severe COVID-19 patients as bacterial superantigens are a well-known trigger of toxic shock syndrome. In fact, a high-affinity motif unique to SARS-CoV-2 was identified that binds the T cell receptor (TCR) and mimics bacterial superantigens (67). Further, MIS-C, a disease emerging as a complication of COVID-19, has also been associated with SARS-CoV-2 as a superantigen (68). MIS-C was observed to rapidly progress to hyperinflammation and shock. Characterization of the TCR repertoire in MIS-C patients revealed enrichment of T cells with specific β-chain variable domain (Vβ) having strong affinity for superantigen-like motif of SARS-CoV-2 spike glycoprotein (69). These TCRβ variable gene 11-2 (TRBV11-2) cells also correlated with MIS-C severity and serum cytokine levels. With viral persistence, these antigens may trigger chronic inflammation, possibly at low level, which may contribute to the pathogenesis of many of the observed post-acute COVID-19 symptoms. These superantigens trigger excessive activation of the adaptive immune system paving way for hyperinflammatory syndrome. It has been suggested that the early phase of COVID-19 infection involves CD8+ T cell inflammation–driven lung damage as a result of the rapid expansion of short-lived effector CD8+ T cells (65). However, this phase may be followed by persistent viral antigen stimulation leading to T cell inactivation, exhaustion, and depletion.

Acute COVID-19 infection has been hypothesized to accelerate several hallmarks of aging (70). Increasing age is often associated with immunosenescence and inflammaging, two terms that entail an exaggerated innate immune and deficient adaptive immune response, in particular to respiratory viral infections (71). While immunosenescence represents the final stage of adaptation of an organism’s immune system to continuous challenges that have occurred throughout life, inflamm-aging refers to the resultant progressive increase in proinflammatory status with aging (72). The long-term sequelae of post-acute COVID-19 suggests an accelerated rate of immunological dysfunction, as seen during immunosenescence and inflammaging. These mechanisms may thus contribute to the persistence of symptoms of a systemic nature. DNA damage, metabolic derangement and increased oxidative stress induced by acute infection can trigger senescence in multiple tissues. Moreover, the release of pathogen-associated molecular patterns (PAMPs) and damage-associated molecular patterns (DAMPs) as a result of direct tissue damage caused by viral invasion activates inflammatory pathways. The viral-induced overt activation of the immune system together with superimposed bacterial infections may overwhelm the immune system resulting in exhaustion and subsequent immunosenescence. This state of chronic systemic inflammation may result in increased and deteriorating age-related conditions including frailty, even among the younger population. In fact, it has been proposed that tissue injury during acute infection may destabilize the homeostatic cellular processes resulting in cellular stress that triggers the buildup of senescent cells across multiple tissue (70). Therefore, despite resolution of acute infection, the continuous release of senescence-associated secretory phenotype (SASP) from the residual senescent cells can initiate a state a chronic inflammation with long-term consequences.

### Viral-Induced Autoimmunity

Viral-induced autoimmunity is another possible contributing mechanism to PASC. SARS-CoV-2 may cause the development of autoreactive T cells and antibodies to endure post-acute infection or even develop post-viral clearance. Recent findings suggest the ability of SARS-CoV-2-specific antibodies to cross-react with mammalian host proteins. Guillain–Barré syndrome is a classic example of such a phenomenon causing post-infectious neuropathy, in which viral-induced antibodies cross-react with self-glycolipids on peripheral nerves. Anti-neuronal autoreactive antibodies have been detected in COVID-19 patients with neurological symptoms indicating the likelihood for the development of autoimmune neurological sequelae, such as autoimmune encephalitis (73–75). A study also identified the ability of a fraction of high-affinity near-germline SARS-CoV-2-neutralizing antibodies to cross-react with self-antigens, including those found in the CNS in murine tissues (76). The identification of target antigens and functional assays for these antibodies will help determine the correlation, if any, between antibody frequency and clinical phenotypes seen in PASC. Further evidence is emerging where different types of autoantibodies have been detected in COVID-19 patients. In a study of 172 patients, 52% were found to have antiphospholipid antibodies of different isotypes (77). Moreover, injection of purified IgG fractions from COVID-19 patients into mice enhanced venous thrombosis (77). This high prevalence of antiphospholipid autoantibodies may help explain the high incidence of thromboembolic events among COVID-19 patients. In another study, 20/29 (68.7%) ICU patients with COVID-19 tested positive for any kind of autoantibodies related to autoimmune rheumatic diseases (78). In a study of 194 COVID-19 patients, a high prevalence of autoantibodies against different proteins was observed (79). These include antibodies against different cytokines, chemokines, complement proteins, immunomodulatory proteins, metalloproteinases endothelial cell surface proteins and others (79). Another study by the same group reported the activation of a B cell repertoire characteristic of autoimmune settings in critically ill COVID-19 patients (80). In fact, approximately 10% of the 987 patients with life-threatening COVID-19 pneumonia demonstrated high titer levels of IgG autoantibodies against type I IFN-α2 and IFN-ω (81). Interestingly, these were characteristic of the severe cases and not detected in the asymptomatic and mild phenotype. In another study, investigators reported that 44% of their cohort of 31 COVID-19 patients tested positive for antinuclear antibodies (82). The pathogenesis of MIS-C has also been associated with multiple autoantibodies (83). In this study, proteome array profiling revealed potentially pathogenic autoantibodies, notably those targeting the glycoprotein endoglin (CD105), MAP2K2 and casein kinases, as an element of the immunopathology of MIS-C. These cumulative evidence may support the contention that SARS-CoV-2 induced autoimmunity likely plays an important role in the pathogenesis of the wide range of acute and chronic manifestations of COVID-19 disease. Further studies are needed to elucidate the pathological role, and the short-term and long-term clinical impact of these reported autoimmune phenomena among SARS-CoV-2 infected individuals.

### Abnormal Immunometabolism and Mitochondrial Dysfunction

Mitochondrial health is pivotal for immune homeostasis and as such pathogens are known to manipulate mitochondrial functions to influence their survival or evade host immunity (84). The state of low-grade inflammation established as a result of continuous antigen stimulation is accompanied by an age-related decline in mitochondrial function (85). For instance, T cell exhaustion is characterized by impaired mitochondrial oxidative phosphorylation and higher rates of glycolysis (86). Acute COVID-19 patients have been reported to exhibit distinct subsets of T cells with mitochondrial dysfunction and increased susceptibility to cell death (87). Peripheral blood mononuclear cells (PBMCs) from COVID-19 patients exhibit increased mitochondrial dysfunction, metabolic alterations and high mitokine levels (88). Thus, arises the possibility that SARS-CoV-2 could either directly or indirectly modulate mitochondrial function. SARS-CoV-1 encodes the open reading frame-9b (ORF-9b) protein that can target mitochondria leading to inhibition of mitochondrial anti-viral signaling proteins (MAVS) and thereby suppressing anti-viral interferon response (89). We have recently reviewed the plausible immune evasion mechanisms, some of which involves targeting the mitochondria, developed by SARS-CoV-2 to promote viral immunopathogenesis (90). Several SARS-CoV-2 proteins such as non-structural proteins (NSPs) 4 and 8, and ORF9c, have also been predicted to interact with mitochondria (91). As such, SARS-CoV-2 has been proposed to hijack the host mitochondria for viral advantage (92). In addition to viral RNA and its transcripts that localize to the mitochondria, ACE2 regulation of mitochondrial function, ORF proteins, mtDNA release and mtDNA-induced inflammasome activation help evade host cell immunity, thereby facilitating virus replication and COVID-19 disease.

This mitochondrial dysfunction may predispose COVID-19 survivors to long-term health consequences. Therefore, it comes as little surprise that patients with metabolic syndromes such as diabetes and obesity, are at heightened risk of severity and mortality from COVID-19. Compromised mitochondrial function and energy insufficiency in COVID-19 patients may lead to metabolic reprograming in the infected cells and a metabolic switch to glycolysis. Consistent with a hypometabolic syndrome, targeted metabolomic studies have reported low levels of several metabolites in patients with chronic fatigue syndrome (93). These patients had impairment in cellular energy generation from almost all sources, including amino acids, lipids, sugars and oxygen, and possibly even in using the limited generated energy (93). A similar mechanism could be responsible for the chronic fatigue observed in patients with long COVID-19.

In addition, the elevated cytokine levels induced by SARS-CoV-2 may facilitate pancreatic beta cell hyperstimulation and insulin resistance leading to exhaustion and subsequent onset of metabolic alterations (94, 95). Recently, abnormalities in beta cell function, insulin resistance and glycometabolic control were documented in a cohort of hospitalized COVID-19 patients (96). Interestingly, these patients did not have any pre-existing history or diagnosis of diabetes. Furthermore, glycemic abnormalities were found to persist for at least 2 months after disease onset in recovered patients suggesting metabolic alterations to play an important role in the context of PASC (96). Abnormal immunometabolism could, therefore, be fueled by the hyperinflammatory state observed in some COVID-19 patients with its associated systemic perturbations including disruption of glycometabolic control, iron dysregulation, reactive oxygen species (ROS) production, oxidative and nitrosative stress.

### Altered Microbiome

Mitochondrial oxidative stress may in turn lead to microbiota dysbiosis contributing to the progression and severity of COVID-19. The microbiome of the gastrointestinal tract is essential for the establishment of immune homeostasis (97). The composition of the gut microbiota was altered in patients with COVID-19, and together with inflammatory cytokines and blood markers reflected disease severity and dysfunctional immune response (98). The dysbiosis of gut microbiota further persisted for up to 30 days after disease resolution which may have a role to play in the persistent symptoms of PASC. Additionally, ACE2 is known to influence the expression of neutral amino acid transporters in the gut (99), thereby regulating the composition of gut microbiota which in turn can modulate local and systemic immune responses (100, 101). Through its impact on intestinal dysbiosis, ACE2 imbalance have been linked to poor outcomes (including higher disease severity and mortality rate) in COVID-19 patients with pre-existing age-related comorbidities (102). Persistence of SARS-CoV-2 in the gut of COVID-19 patients was also recently shown to direct zonulin-dependent loss of gut mucosal barrier in MIS-C patients (103) suggesting a possible mechanism by which prolonged presence of SARS-CoV-2 in the gut paves way to intestinal dysbiosis with long-term consequences. Moreover, dysbiosis has been implicated in the autoimmune settings (104).

### Imbalance in Renin-Angiotensin System

The renin-angiotensin system (RAS) plays a key role in maintaining the physiological balance of the body, thereby influencing the function of multiple organ systems (105). ACE2 catalyzes the conversion of angiotensin II (Ang II) to angiotensin (1-7). SARS-CoV infections are accompanied by a decline in ACE2 expression, subsequent increase in Ang II levels and potential overactivation of RAS (106). However, the ACE2/Ang (1-7)/MAS axis counteracts the classical RAS pathway. Elevated plasma Ang II levels have been associated with lung injury and pathogenesis of critically ill COVID-19 patients (107). The imbalance between ACE2/angiotensin axis and RAS have also been implicated in multi-organ injury associated with COVID-19 (108). RAS imbalance in a swine model induced a pathophysiological phenotype with systemic effects, comprising of diffused alveolar damage, disturbed lung perfusion, increased coagulation, reduced blood oxygenation, increased pulmonary arterial pressure and acute tubular necrosis, that shared similarities with clinical COVID-19 presentation (109). The RAS imbalance in the acute COVID-19 phase may cause substantial end-organ damage in lung, heart, kidney and small intestine amongst others, paving way for long-term health outcomes in these patients.

COVID-19 infection thus, appears to jeopardize the host immune system enabling it to unleash its ongoing inflammatory damage long after the infection subsides. It is highly possible that an activated inflammatory process as a result of COVID-19 infection constitutes the primary underlying pathophysiological mechanism for these chronic symptoms. This is supported by the fact that the pathology in severe COVID-19 patients is frequently associated with a hyperinflammatory cytokine storm. These inflammatory cytokines can cause long-term damage, not only to the lungs but also to the distant organs, resulting in significantly widespread burden of the disease. The persistence of these symptoms post recovery suggests potentially considerable burden of inflammatory disease in COVID-19 patients and creates an urgent need for larger and longer-term cohort studies to follow-up on the long-term health effects of COVID-19. In summary, some of the underlying immune mechanisms that are predicted to contribute towards the long-term consequences of COVID-19 infection include viral infection-related tissue damage, collateral damage from hyperinflammation, immune exhaustion, post-viral autoimmunity, dysregulated immunometabolism and microbial dysbiosis. Therefore, multiple disease processes with varying degrees of overlap may contribute to the long-term sequelae of COVID-19.

## Long-Term Multi-Organ Clinical Sequelae of COVID-19

As previously mentioned, patients with PASC are anticipated to experience slow or incomplete recovery with varying degrees of physical, cognitive, mental/psychosocial and social health problems (110). The underlying abnormalities across multiple organ systems has led to the recognition of PASC as a complex systemic disease that profoundly affects the lives of its patients. The current evidence on the long-term multi-organ impairments observed in PASC is presented here corresponding to the organ systems involved (**Figure 2**). **Table 1** summarizes these multi-organ clinical sequelae of COVID-19.

**Table 1:** A summary of the clinical sequelae of COVID-19 (32, 111, 112).

Organ Systems	Clinical Manifestations	Pathological features	Potential Underlying Biology
Respiratory system	Chronic cough Shortness of breath (dyspnea), breathlessness Chest pain Reduced exercise capacity Acute respiratory diseases Fibrotic lung disease Bronchiectasis Pulmonary vascular disease	Congestive lungs with alveolitis Ground glass opacities Pulmonary lesions Mononuclear inflammatory cell (Monocyte and macrophage) and fibrinous exudate Inflammatory edema in respiratory mucosa and alveolar wall Platelet-fibrin thrombi Necrotising bronchiolitis, diffuse alveolar damage (DAD), hyaline membrane formation	Direct viral invasion *via* ACE-2 expression in the upper airway(goblet and ciliated epithelial cells), lower respiratory tract epithelium (type II alveolar), and pulmonary vasculature (arterial smooth muscle), and endothelial cells Residual virus in lungs post recovery Cytokine storm Activation of the complement system Microthrombi and macrothrombi formation
Cardiovascular system	Chest pain Palpitations Ventricular dysfunction Myocardial injury Myocarditis Cardiomyopathy Cardiac arrhythmias Myocardial ischemia Thromboembolism	Cardiac Increased troponin levels Low-grade myocardial inflammation Hypertrophied cardiomyocytes with inflammatory infiltrates Focal edema Interstitial hyperplasia Fibrosis Degeneration, necrosis and signs of lymphocytic myocarditis Hematologic Edematous changes in alveolar capillaries Fibrin thrombi Perivascular inflammatory infiltrates	Direct viral invasion *via* ACE-2 receptor in cardiac tissue (pericytes, endothelial cells, cardiomyocytes, cardiofibroblasts, and epicardial adipose cells, and vascular cells) Cytokine storm Hyperinflammation Endothelial dysfunction Leucocyte infiltration Formation of microvascular thrombosis
Nervous system	Fatigue Myalgia Anxiety Depression PTSD Sleep disorders Headaches Taste and smell impairment (ageusia and anosmia) Cognitive impairment (brain fog) Mood swings Seizures Ischemic or hemorrhagic stroke Encephalitis	Brain lesions Hyperemia, edema and neuronal degeneration Demyelination Acute hypoxic ischemic injury	Proposed SARS-COV-2 viral invasion by breaching blood–brain barrier or through olfactory nerves Hypoxia Cytokine storm Hyperinflammation Coagulation abnormalities Endothelial dysfunction
Urinary system/Kidney	Acute kidney injury Albuminuria Proteinuria Hematuria	Diffuse proximal tubule injury Protein exudate in balloon cavity and thrombus in capillaries Non-specific fibrosis with lymphocytic infiltrates Acute tubular necrosis	Direct viral invasion *via* positive ACE-2 expression in kidney tissue (proximal tubule epithelial cells, glomerular endothelial cells, podocytes and kidney vasculature) Cytokine storm Systemic hypoxia Activation of complement components (C5b-9) Abnormal coagulation
Digestive system/Liver	Acute liver injury Cholestasis Elevated serum liver biomarkers (aspartate aminotransferase (AST), alanine aminotransferase (ALT), bilirubin)	Hepatic cell degeneration Multi-focal necrosis, indicative of cirrhosis Biliary plugs in the small bile duct Atypical lymphocytic infiltration in the portal tract Increased number of portal veins Activated Kupffer cells Smooth muscle fragmentation of portal vein	Direct viral invasion *via* ACE-2 expression in the hepatobiliary system (cholangiocytes, hepatocytes and bile duct cells) Systemic inflammation Hypoxia Drug-induced damage Coagulation abnormalities
Digestive system/Gastrointestinal tract	Diarrhea Decreased appetite Nausea/Vomiting Abdominal pain Gastrointestinal bleeding Anorexia	Stenosis of small intestine Segmental dilatation Degeneration, necrosis and shedding in the gastrointestinal mucosa Inflammatory infiltrates	Direct viral invasion *via* ACE-2 expression in digestive tract (small intestinal enterocytes) Alteration of intestinal microbial flora Cytokine storm
Reproductive system/Testis	Orchitis Infertility Sterility	Leucocyte infiltration Edematous testicular cells Destruction of the seminiferous tubules Reduced spermatogenesis	Direct viral invasion *via* positive ACE-2 and TMPRSS2 expression in testicular cells Hyperinflammation
Dermatological system/Skin	Hair loss Erythematous rash Dermatitis Pseudo-chilblains on fingertips and toes Urticaria Chicken pox-like vesicles*	Vasculitis Dermatological lesions in trunk, hands and feet Perivascular inflammatory infiltrates in the superficial dermis with extravasation of red blood cells and intraluminal thrombi Capillary thrombosis with diffuse hemorrhage Parakeratosis, acanthosis, dyskeratotic keratinocytes, necrotic keratinocytes, acantholytic clefts along with lymphocytes satellitisms	Direct viral invasion *via* positive ACE-2 expression in endothelium, stratum basale, sebaceous and eccrine cells

### Respiratory Impairments

Respiratory complications are not unusual in PASC patients considering some degree of impairment and functional limitation in lung function during the course of COVID-19. Pathological evidence of the persistence of residual virus in the lungs after three consecutive negative PCR test results suggests the likelihood of the SARS-CoV-2 virus or viral particles to persist in the lung despite a negative nasopharyngeal swab (35). The atypical pneumonia and acute respiratory distress syndrome (ARDS) associated with COVID-19 can cause lasting damage to the lung alveoli through irreversible scarring or fibrosis. This may lead to long-term breathing problems as well as the development of pulmonary fibrosis (19). Several studies have shown varying degrees of structural and functional pulmonary abnormalities long after recovery from the acute illness among COVID-19 patients. For example, in a study on 55 COVID-19 survivors three months after recovery, 35 (64%) of them showed SARS-CoV-2 related persistent symptoms and 39 (71%) of them showed different degrees of radiological and physiological lung abnormalities (113). In another study, half of the enrolled patients exhibited decreased lung diffusing-capacity, lower respiratory muscle strength, and lung imaging abnormalities in the early convalescence phase (30 days after hospital discharge) (114). A longitudinal study of patients with COVID-19 pneumonia reported residual lung computed tomography (CT) abnormalities with predominantly ground-glass opacity in 94% of the discharged patients (115). These abnormal lung CTs were not restricted to the hospitalized, severe cases, but were also found in asymptomatic patients (116). Similar to patients with severe COVID-19 disease, those with moderate disease who had two consecutive negative RT-PCR tests after treatment showed residual peripheral pulmonary lesions across all lobes (117). The presence of pulmonary histopathological changes in COVID-19 patients (111) further supports the manifestation of chronic lung tissue damage resulting in long-term respiratory symptoms. Similarly, longitudinal studies of SARS and MERS patients have also identified long-term lung damage and lung functional abnormalities in roughly a third of the survivors (25, 118). Despite these abnormalities in imaging and functional parameters, the long-term clinical significance of these findings needs further elucidation.

In addition to direct viral invasion *via* ACE2 expression in the upper airway (goblet and ciliated epithelial cells), lower respiratory tract epithelium (type II alveolar), pulmonary vasculature (arterial smooth muscle), and endothelial cells, immunological damage has been implicated in the development of ARDS and subsequent long-term respiratory impairments. Accelerated lung fibrosis in some COVID-19 patients after the resolution of infection (119) may be triggered by elevated levels of pro-inflammatory cytokines, in particular IL-6 and TGF-β, which are implicated in the pathogenesis of lung fibrosis (120, 121). The increased pulmonary microthrombi and macrothrombi formation in COVID-19 patients (122) may also contribute to the long-term respiratory sequelae.

### Cardiovascular Impairments

Cardiac complications, in particular, arrhythmias and myocardial injury, have been associated with COVID-19. Echocardiographic and magnetic resonance imaging of the heart tissue, months after COVID-19 recovery, demonstrated evidence of myocardial damage, even in those experiencing mild symptoms, increasing the patient risk of heart failure and other future complications (123). In a prospective observational cohort study, 78% of the COVID-19 patients who had recently recovered from the illness demonstrated abnormal cardiovascular magnetic resonance (CMR) findings and 60% had ongoing myocardial inflammation (124). The prevalence of these abnormalities 71 days after the original COVID-19 diagnosis was independent of pre-existing conditions, severity of the disease, presence of cardiac symptoms and time from original diagnosis. Furthermore, in a cohort of competitive athletes who had recovered from COVID-19, majority without any reported symptoms and none requiring hospitalization, CMR imaging provided evidence of myocarditis and prior myocardial injury in 15% and 31% of the cohort, respectively (125). These observations of cardiovascular involvement even in patients with mild acute symptoms, mostly home-based recovery and relatively lower prevalence of pre-existing cardiovascular conditions, indicate increased risk of significant cardiac complications in the early convalescent stage and the long-term period post-acute COVID-19 disease. Direct viral invasion *via* ACE2 receptor in cardiac tissue (pericytes, endothelial cells, cardiomyocytes, cardiofibroblasts, and epicardial adipose cells, and vascular cells), hyperinflammation, endothelial dysfunction affecting the integrity of the myo- and peri-cardium may perpetuate cardiovascular damage in COVID-19 survivors. Further, dysregulation of RAS may also create a persistent cardiometabolic demand in recovered patients (126). The presence of histopathological changes in the heart of COVID-19 patients (111) reinforces the manifestation of cardiac sequelae in cpost-acute COVID-19.

Predisposition to thrombotic complications is another feature of COVID-19 which causes diffuse intravascular coagulation and thromboembolic events involving different organs. Despite limitations of small sample size, inadequate follow-up and variability in outcomes, the rate of venous thromboembolism (VTE) in post-acute COVID-19 may be estimated to be less than 5% (32). Endothelitis can also potentially contribute to persistent damage to other organs, including the lungs, brain, liver and kidneys (127). The hypercoagulable and hyperinflammatory states associated with COVID-19 (128) may explain the risk of thrombotic complications in patients who have recovered from acute infection.

### Neurological Impairments

Neurological deficits, including strokes, seizures and Guillain-Barre syndrome, have been reported in more than one third (36.4%) of COVID-19 patients (21). Affected patients may also be susceptible to developing cognitive decline after overcoming the primary COVID-19 infection, in particular Alzheimer’s disease. The persistence of cognitive impairment and motor deficits in a third of the discharged patients reinforces the risk of developing long-term neurological consequences (129). COVID-19 has thus, been linked with the risk of developing Parkinson’s disease and Alzheimer’s disease (130).

The continued detrimental effects of COVID-19 on the central and autonomic nervous system could be the result of direct viral encephalitis, systemic inflammation, peripheral organ dysfunction (liver, kidney, lung), and/or cerebrovascular changes (131). Immunosenescence and associated inflammaging may also have a role to play in the persistence of neuropathology. These abnormalities may either aggravate a pre-existing neurological disorder or trigger a new one. Systemic inflammation that could promote cognitive decline and neurodegenerative diseases supports the likelihood of neurodegeneration in COVID-19 survivors. For instance, it is known that ARDS patients often experience subsequent cognitive impairment, executive dysfunction, and reduced quality of life, that can last for months after hospital discharge (132). Neuropsychiatric sequelae that lasted for months post recovery in the past epidemics such as SARS, MERS and influenza, may also threaten the cognitive health, day-to-day functional status, and overall health and well-being of COVID-19 survivors.

### Psychological Impairments

COVID-19 can also cause severe psychological disorders. For instance, the admission of patients with severe symptoms to the intensive care unit (ICU) with mechanical ventilation makes them more likely to develop ICU-acquired neuro-cognitive or psychological illnesses such as anxiety, depression and post-traumatic stress disorder (PTSD). Although it is still too early to predict the full spectrum of long-term psychological consequences of COVID-19, much can be learned from related viruses, such as the SARS-CoV-1 virus. In addition to fatigue, sleep deprivation and cognitive/mental health impairments such as delirium, brain fog, memory loss, hallucination, confusion, depression and anxiety are experienced by COVID-19 recovered patients (133). However, their underlying pathology is yet to be understood.

### Gastrointestinal and Hepatic Impairments

Many COVID-19 patients experience an array of gastrointestinal (GI) symptoms, mainly diarrhea, nausea and anorexia (134). In a systematic review that included 43 studies with over 18,000 patients, diarrhea was the most common symptom which was reported in 11.5% of patients (135). Nausea and vomiting were experienced by 6.3% and abdominal pain by 2.3% of patients. In another meta-analysis of 35 studies, the pooled prevalence of GI symptoms was 15%, with nausea or vomiting, anorexia and diarrhea being the most common (136). Furthermore, the pooled prevalence of liver function abnormalities was 19% (136). The severity of the underlying COVID-19 disease also seemed to correlate with the degree of abdominal pain and hepatic dysfunction. However, the prevalence and the nature of GI and hepatic manifestations among PASC patients are not yet clear. Gastrointestinal sequelae were observed in 44% of 117 COVID-19 patients at 90 days post discharge (137). Loss of appetite (24%), nausea (18%), acid reflux (18%) and diarrhea (15%) were the most commonly reported gastrointestinal symptoms in this study.

Moreover, even patients who did not experience GI symptoms exhibited stool positivity for SARS-CoV-2, which remained active despite viral clearance from the airways (38). Thus, despite the absence of any GI symptoms during and after recovery from the initial infection, viral persistence in the GI tract is a possible explanation for active and prolonged ‘quiescent’ gut infection. In this regard, a recent study demonstrated the involvement of prolonged viral presence in the gut in the pathogenesis of MIS-C through zonulin-dependent loss of gut mucosal barrier and subsequent development of hyperinflammation (103).

### Metabolic Impairments

Extreme fatigue and incapability to perform daily life activities is a common complaint among COVID-19 survivors. They share features with chronic fatigue syndrome (CFS) encountered after SARS, MERS, and community-acquired pneumonia. Similar to the defects in lipid and glucose metabolism identified in SARS survivors’ years after clinical recovery (126, 138), new-onset diabetes and associated diabetic ketoacidosis have also been observed in COVID-19 patients (139–141), suggesting lasting metabolic impairments in PASC. Increased prevalence of hyperglycaemia has also been reported among COVID-19 patients (96, 142, 143). Furthermore, an increased rate of new-onset hyperglycaemia was noted in hospitalized COVID-19 patients with nearly 35% of 551 patients exhibiting persistent hyperglycaemia for up to 6 months (96). These patients with new-onset hyperglycaemia also exhibited a higher clinical score and required a longer in-hospital stay. Thus, new-onset hyperglycaemia in COVID-19 patients may predispose patients to increased risk of poor clinical outcomes and long-term hyperglycaemia.

### Post-ICU Complications

Patients treated for critical COVID-19 in the ICU often need long periods of ventilation, neuromuscular blockade and sedation (144, 145). In addition, they suffer physical, cognition, and mental impairments during ICU stay as well as post discharge (146). Patients who survive critical COVID-19 experience post-intensive care syndrome (PICS) which often occurs after prolonged critical illnesses, such as COVID-19-associated ARDS or severe sepsis, and it involves persistent inflammation, immunosuppression and chronic organ dysfunction. Metabolic alterations during critical illness, immobility, microvascular ischemia and injury may contribute to the pathophysiology of PICS (146). Substantial morbidity and mortality have been reported to accompany PICS. Moreover, the prevalence and severity of PICS among COVID-19 survivors may be greater than in general sepsis cohorts because the pandemic has overwhelmed ICUs in many parts of the world and hindered optimal care (30, 145, 147–150).

## Post-COVID-19 Assessment and Rehabilitation Programs

The heterogeneity in the clinical presentation of PASC patients prompts continuous monitoring of the course of symptoms and their functional impact on the patient. A “Post-COVID-19 Functional Status (PCFS) scale” was proposed to track the full spectrum of functional outcomes over time in COVID-19 patients following hospital discharge (151). Provocation studies with cognitive, postural and physical challenges would help clarify the biological abnormalities that could causally be connected to the post-COVID symptoms. For instance, the 10-minute NASA Lean Test (NLT) is a simple and clinically useful point-of-care method. This may aid in the early diagnosis of PASC as well as help guide the management and treatment of orthostatic intolerance. A comprehensive assessment of five parameters: ADLs, respiratory function, physical function, cognitive function and quality of life is recommended to assess the functional limitations post-COVID-19 (18). A multidisciplinary rehabilitation approach is essential to ensure the continuum of care for these patients enabling them to achieve a gradual but complete recovery. The Stanford Hall consensus statement for post-COVID-19 rehabilitation has recognized and classified the requirements of multidisciplinary rehabilitation post COVID-19 illness into the following domains – pulmonary, cardiac, sport and exercise medicine (SEM), psychological, musculoskeletal, neurorehabilitation and general medical (152). Innovative approaches including virtual rehabilitation programs (such as video-linked and online classes, home education booklets, telephonic support) are likely to become the norm ahead.

## Future Perspectives

Literature on post-acute sequelae of COVID-19 is fast evolving. Nevertheless, there is a need for better comprehension of the incidence and prevalence of the heterogenous disease manifestations. Further, prospective studies are encouraged to include acute COVID-19 patients and follow-up at different time points post disease onset, and across patient populations with varying clinical characteristics. A virtual workshop convened by the National Institute of Allergy and Infectious Diseases, in collaboration with other Institutes and Centers of the National Institutes of Health identified key knowledge gaps in the area of PASC (153). Considering the diverse manifestations of the disease, the full clinical spectrum of symptoms experienced by these patients over time need to be catalogued to develop a better understanding of their underlying pathology to be able to effectively design clinical trials with improved treatment options. This would also further aid in the characterization of the various phenotypes of PASC and the risk factors associated with their development.

With regards to the pathophysiology of PASC, there are key gaps in knowledge. Direct tissue damage caused by the virus and dysregulated immune response are the two predominant pathophysiological mechanisms that drive the diverse clinical manifestations. However, the extent of damage caused by each is yet to be clearly understood. Future studies need to investigate the site, magnitude and duration of viral infection in association with the type and timing of multi-organ sequelae. Viral mutations are another topic of significance at present and the prevalence of PASC across the various SARS-CoV-2 mutants need to be explored. The potential role of coagulopathy and vasculitis in the pathophysiology of PASC was also identified as an area that needs to be explored (153). At the same time, the impact of re-infection on the development of PASC warrants investigation.

With growing international interest and increased research support, advances in understanding the pathophysiology would lead to effective therapy, in terms of improved diagnostic tests and approved treatment strategies, providing some hope to these patients. The full spectrum of these long-term health consequences is not yet fully understood. Therefore, there is an urgent need for the establishment of outpatient post–COVID-19 clinics to follow-up on COVID-19 survivors, to better understand and treat these chronic symptoms and thus prevent the perpetuation of chronic ill health in those that have recovered from acute COVID-19 infection. Furthermore, the contributing mechanisms may vary from person to person reinforcing the need for appropriate treatment for each long-hauler based on their exhibited symptoms and underlying disease profile. Improved management in the months following hospital discharge should focus on 1) the identification of any new physical, mental or cognitive problems and subsequent referral for appropriate treatment, 2) reviewing the long-term medications and adjusting their dosages, and 3) constant evaluation for conditions that result in hospitalization, including heart failure, renal failure and infections. Clinical trials assessing the efficacy of anti-inflammatory therapeutics hold potential in discovering key immune modulators that could benefit these chronic immune-mediated COVID-19 symptoms. Considering the diversity of these chronic symptoms with possibly variable underlying causes, a multidisciplinary approach with contributions from different medical fields, such as immunology, respiratory medicine, cardiovascular biology, neurology, liver medicine, renal medicine, rheumatology and endocrinology, may help in better understanding the biological mechanisms that drive the pathology behind PASC.

## Conclusions

Post-acute sequelae of COVID-19 is an emerging public health disease that could lead to a huge global burden. The combination of initial SARS-CoV-2 viral insult together with ongoing abnormalities in the host immunoregulatory systems is thought to pave the way for the clinical sequelae of COVID-19. More research is needed to understand the natural course of PASC, its associated pathology, the underlying mechanisms, and the potential treatments and rehabilitation programs.

## Author Contributions

RR: Data curation, formal analysis, writing – original draft, writing – review & editing. TK, QH, RH, and IT: Conceptualisation, formal analysis, supervision, validation, writing – review & editing. All authors contributed to the article and approved the submitted version.

## Funding

We would like to acknowledge the support of COVID-19 research grant (CoV19-0307); Seed grant (Grant code: tel:2001090275); collaborative research grant (Grant code: tel:2001090278 and 2001090283); Sandooq Al Watan Applied Research & Development grant (SWARD-S20-007); Al Jalila Foundation Seed Grant (AJF202019); and Prince Abdullah Ben Khalid Celiac Disease Research Chair, under the Vice Deanship of Research Chairs, King Saud University, Riyadh, Kingdom of Saudi Arabia to RH, University of Sharjah, UAE.

## Conflict of Interest

The authors declare that the research was conducted in the absence of any commercial or financial relationships that could be construed as a potential conflict of interest.
